# Enhancing one-year mortality prediction in STEMI patients post-PCI: an interpretable machine learning model with risk stratification

**DOI:** 10.3389/frai.2025.1618492

**Published:** 2025-08-22

**Authors:** Wenqiang Li, Dongdong Yan, Wei Hu, Xiaoling Su, Zheng Zhang

**Affiliations:** ^1^The First Clinical Medical School, Lanzhou University, Lanzhou, China; ^2^Department of Cardiology, Qinghai Provincial People’s Hospital, Xining, China; ^3^Heart Center, The First Hospital of Lanzhou University, Lanzhou, China; ^4^Key Laboratory for Cardiovascular Diseases of Gansu Province, Lanzhou, China; ^5^Cardiovascular Clinical Research Center of Gansu Province, Lanzhou, China

**Keywords:** one-year mortality, ST-segment elevation myocardial infarction, machine learning, prediction model, data imbalance, data process method

## Abstract

**Background:**

ST-elevation myocardial infarction (STEMI) poses a significant threat to global mortality and disability. Advances in percutaneous coronary intervention (PCI) have reduced in-hospital mortality, highlighting the importance of post-discharge management. Machine learning (ML) models have shown promise in predicting adverse clinical outcomes. However, a systematic approach that combines high predictive accuracy with model simplicity is still lacking.

**Methods:**

This retrospective study applied three data processing and ML algorithms to address class imbalance and support model development. ML models were trained to predict one-year mortality in STEMI patients post-PCI, with performance evaluated using accuracy, sensitivity, precision, F1-score, area under the receiver operating characteristic curve (AUROC), and the area under the precision-recall curve (AUPRC).

**Results:**

We analyzed data from 1,274 patients, incorporating 46 clinical and laboratory features. Using the Random Forest (RF) algorithm, we achieved an AUROC of 0.94 (95% confidence interval (CI): 0.90–0.98), an AUPRC of 0.44 (95% CI:0.15–0.76) in the internal validation set, identifying five key predictors: cardiogenic shock, creatinine, NT-proBNP, diastolic blood pressure, and left ventricular ejection fraction. By integrating risk stratification, the model’s performance improved, achieving an AUROC of 0.97 (95% CI: 0.96–0.99) and an AUPRC of 0.74 (95% CI: 0.60–0.84).

**Conclusion:**

This study highlights the feasibility of constructing accurate and interpretable ML models using a minimal set of predictors, supplemented by risk stratification, to improve long-term outcome prediction in STEMI patients.

## Introduction

Acute myocardial infarction (AMI) remains a leading cause of morbidity and mortality worldwide, with ST-elevation myocardial infarction (STEMI) representing the most severe form, accounting for 50–60% of AMI cases in contemporary registries (Gaudino et al., 2023; Miller, 2020; Cederström et al., 2024). Advances in emergency care systems, the establishment of chest pain centers, and the widespread implementation of percutaneous coronary intervention (PCI) have significantly reduced in-hospital mortality, now reported to be between 4 and 8% (Khera et al., 2021; Liu et al., 2021). Consequently, optimizing post-discharge management for STEMI survivors has become increasingly important, with a strong emphasis on the early identification of high-risk individuals to enhance long-term outcomes.

Machine learning (ML) has shown substantial promise in risk stratification and outcome prediction across various clinical domains (Wang et al., 2021; Oliveira et al., 2023; Khera et al., 2024). Compared to traditional statistical approaches, ML models offer enhanced predictive accuracy and individualized risk assessment (Mohd Faizal et al., 2021; Stephan et al., 2025). However, despite the increasing availability of predictive models, standardized approaches for selecting the optimal model remain limited (Ogunpola et al., 2024; Radwa et al., 2024). The area under the receiver operating characteristic curve (AUROC) is commonly used to evaluate model performance (Lee et al., 2020; Payrovnaziri et al., 2019; Fukumoto et al., 2021). However, when multiple models demonstrate similarly high AUROC values, it becomes challenging to determine a clear winner. Furthermore, as the complexity of medical data continues to grow—along with the number of candidate models and input variables—developing models that balance predictive accuracy with clinical simplicity has become a key challenge.

This study aimed to construct a predictive model tailored to the characteristics of the dataset by selecting appropriate data processing methods and ML algorithms. Our objective was to maximize predictive performance while minimizing model complexity. The key contributions of this work include: (1) demonstrating that different data processing strategies yield no statistically significant differences in model performance; (2) showing that in highly imbalanced datasets where AUROC lacks discriminative capacity, the area under the precision-recall curve (AUPRC) provides a more informative metric (Zhou et al., 2021; Zheng et al., 2024); (3) proposing bootstrap testing as a viable alternative to DeLong’s test for comparing area under curve values; and (4) constructing a high-performing predictive model using a minimal set of readily available clinical variables, with further gains achieved through risk stratification.

Additionally, this study aimed to develop predictive models for one-year mortality in patients with STEMI post-PCI, utilizing the Shapley Additive Explanations (SHAP) method for model interpretability. We also developed a web-based application that enables clinicians to predict individual patient outcomes by inputting the required model variables, thereby facilitating personalized risk assessment.

## Materials and methods

### Study population

This study involved a retrospective analysis of patients admitted to the First Hospital of Lanzhou University in Gansu Province, China, between January 1, 2019, and December 31, 2020. All consecutive hospitalized patients diagnosed with STEMI and treated with PCI during this period were screened for eligibility. STEMI was diagnosed based on criteria established by the European Society of Cardiology (ESC) Association (Huang et al., 2025; Razavi et al., 2025). Inclusion criteria were: (1) a confirmed diagnosis of STEMI, (2) age ≥18 years, (3) receipt of PCI, and (4) availability of complete clinical data. Details of the inclusion and exclusion process are provided in Supplementary Figure S1.

Follow-up information was obtained through structured telephone interviews conducted by a dedicated follow-up center, supplemented by outpatient clinical assessments.

### Data collection

Clinical and laboratory data for patients with STEMI were extracted from electronic medical records, with one-year all-cause mortality defined as the primary outcome. Detailed clinical and laboratory variables are presented in Table 1. For each patient, the initial set of laboratory test results obtained upon hospital admission—including blood samples—was utilized for analysis. A two-step approach was employed to address missing data: variables with more than 20% missing values were excluded, while those with less than 20% missing values were imputed using the IterativeImputer method to minimize bias.

**Table 1:** Comparison of baseline characteristics of the study population.

Variables	2019 STEMI		2020 STEMI	
	Survived (*n* = 634)	Deceased (*n* = 38)	Survived (*n* = 581)	Deceased (*n* = 21)
Age (y)	60.46 ± 10.9	65.53 ± 12.61**	59.45 ± 10.81	66.76 ± 13.08
Gender (%)	553 (87.22)	31 (81.58)	500 (86.01)	18 (85.71)
BMI (Kg/m^2^)	23.68 ± 3.44	23.44 ± 3.93	24 ± 3.51	22.52 ± 2.96
TIT (h)	37.59 ± 73	43.31 ± 65.06	33.41 ± 92.18	35.51 ± 61.86
Medical history				
T2DM (%)	111 (17.51)	13 (34.21)*	104 (17.9)	7 (33.33)
HTN (%)	297 (46.85)	20 (52.63)	228 (39.24)	10 (47.62)
HLD (%)	21 (3.31)	0 (0)	181 (31.15)	8 (38.1)
PAD (%)	27 (4.26)	4 (10.53)	17 (2.93)	4 (19.05)**
SH (%)	398 (62.78)	20 (52.63)	242 (41.65)	11 (52.38)
CAD (%)	42 (6.62)	4 (10.53)	36 (6.2)	4 (19.05)*
BH (%)	7 (1.1)	0 (0)	14 (2.41)	3 (14.29)*
Baseline vital signs				
SBP (mmHg)	116.87 ± 22.95	99.89 ± 27.83***	113.81 ± 25.61	102.57 ± 33.23
DBP (mmHg)	76.55 ± 14.99	63.21 ± 17.19	72.14 ± 15.75	64.81 ± 20.45
HR (beats/min)	82 (70, 91)	88 (66.5, 99.5)	78 (67, 89)	94 (79, 102)***
Baseline laboratory values				
CREA (umol/L)	72 (64, 84)	111 (74, 143)***	70 (61, 80)	91 (66, 129)**
UA (umol/L)	351 (286, 413)	407 (323, 472)**	344 (286, 412)	423 (339, 495)*
RBG (mmol/L)	6.77 (5.62, 8.98)	10.27 (6.98, 15.74)***	6.88 (5.67, 9.04)	12.31 (6.95, 20)***
LDLC (mmol/L)	2.93 ± 0.82	2.71 ± 0.78	2.99 ± 0.88	2.77 ± 1.00
HCT (%)	45.13 ± 5.46	44.84 ± 6.48	44.84 ± 6.12	42.59 ± 6.28
NEUT (10^9^ /L)	8.05 ± 3.17	10.01 ± 3.31***	9.10 ± 8.31	11.15 ± 5.22
LYMPH (10^9^ /L)	1.49 ± 0.74	1.54 ± 1.08	1.66 ± 1.93	1.47 ± 1.05
NLR	7.12 ± 5.03	9.21 ± 5.85*	8.09 ± 10.30	11.05 ± 9.98
HGB (g/L)	153.11 ± 18.03	149.95 ± 21.69	152.21 ± 18.08	144.24 ± 21.33
PLT (10^9^ /L)	197.31 ± 73.34	195.89 ± 65.93	184.17 ± 60.74	176.76 ± 54.76
MYO (ng/ml)	447.31 ± 338.41	576.96 ± 351.92	425.42 ± 337.39	609.76 ± 361.82
CKMB (ng/ml)	150.06 ± 167.02	176.41 ± 179.83*	154.83 ± 174.19	169.19 ± 169.92
TNI (ng/ml)	6.63 ± 8.68	9.19 ± 9.89	7.41 ± 9.12	9.33 ± 9.59
NT-proBNP (pg/ml)	480 (186, 1,435)	4,305 (1,260, 8,715)***	473 (158, 1,340)	3,093 (566, 4,860)***
Echocardiographic findings				
LAD (cm)	3.23 ± 0.37	3.25 ± 0.45	3.3 (3.1, 3.5)	3.3 (2.7, 3.5)
LVEF (%)	51 (47, 57)	45 (39, 51)***	53 (48, 57)	44 (30, 45)***
LVEDV (ml)	120 (104, 142)	120 (105, 162)	126 (106, 147)	140 (121, 178)*
LVESV (ml)	57 (47, 70)	65 (56, 92)**	58 (49, 72)	85 (65, 99)***
Discharge medication				
BB (%)	459 (72.4)	21 (55.26)*	464 (79.86)	8 (38.1)***
ACEI (%)	286 (45.11)	6 (15.79)***	281 (48.36)	3 (14.29)**
ARB (%)	29 (4.57)	2 (5.26)	37 (6.37)	1 (4.76)
ARNI (%)	12 (1.89)	5 (13.16)**	27 (4.65)	2 (9.52)
SGLT2i (%)	2 (0.32)	0 (0)	23 (3.96)	1 (4.76)
STAT (%)	631 (99.53)	33 (86.84)***	576 (99.14)	14 (66.67)***
ASA (%)	631 (99.53)	33 (86.84)***	571 (98.28)	14 (66.67)***
TICA (%)	433 (68.3)	22 (57.89)	457 (78.66)	10 (47.62)**
CLOP (%)	218 (34.38)	11 (28.95)	120 (20.65)	5 (23.81)
PPI (%)	602 (94.95)	32 (84.21)*	437 (75.22)	13 (61.9)
Complications				
MB (%)	4 (0.63)	3 (7.89)**	1 (0.17)	5 (23.81)***
VF (%)	21 (3.31)	7 (18.42)***	3 (0.52)	6 (28.57)***
AF (%)	25 (3.94)	7 (18.42)**	4 (0.69)	2 (9.52)*
CS (%)	7 (1.1)	18 (47.37)***	2 (0.34)	14 (66.67)***

Compared with Survived, *p < 0.05; **p < 0.01; ***p < 0.001 For normally distributed continuous variables, data are presented as mean ± standard deviation. For variables with abnormal distributions, data are expressed as median values (Q1, Q3). Categorical values are presented as count (percentage). The eGFR was calculated based on the first available serum creatinine during the first 24 h after admission. TIT, total ischemic time; CAD, coronary artery disease; HTN, hypertension; T2DM, type 2 diabetes mellitus; HLD, hyperlipidemia; PAD, peripheral arterial disease; SH, smoking history; BH, bleeding history; BMI, body mass index; SBP, systolic blood pressure; DBP, diastolic blood pressure; HR, heart rate; CREA, serum creatinine; UA, uric acid; RBG, random blood glucose; LDL-C, low-density lipoprotein cholesterol; eGFR, estimated glomerular filtration rate; HCT, hematocrit; NEUT, neutrophil count; LYMPH, lymphocyte count; NLR, neutrophil-to-lymphocyte ratio; HGB, hemoglobin; PLT, platelet count; CRP, c-reactive protein; MYO, myoglobin; CK-MB, creatine kinase-mb; TNI, troponin I; NT-proBNP, n-terminal pro b-type natriuretic peptide; HbA1c, hemoglobin a1c; LAD, left atrial diameter; LVEF, left ventricular ejection fraction; LVEDV, left ventricular end-diastolic volume; LVESV, left ventricular end-systolic volume; BB, beta-blockers; ACEI, angiotensin-converting enzyme inhibitors; ARB, angiotensin II receptor blockers; ARNI, angiotensin receptor-neprilysin inhibitors; SGLT2i, sodium-glucose cotransporter-2 inhibitors; STAT, statins; ASA, aspirin; TICA, ticagrelor; CLOP, clopidogrel; PPI, proton pump inhibitors; VF, ventricular fibrillation; AF, atrial fibrillation; CS, cardiogenic shock.

A total of 46 variables were included in the final dataset, comprising demographic information, cardiovascular history, and laboratory measurements. These features included: gender, age, total ischemic time (TIT), coronary artery disease (CAD), hypertension (HTN), type 2 diabetes mellitus (T2DM), hyperlipidemia (HLD), peripheral arterial disease (PAD), smoking history (SH), bleeding history (BH), body mass index (BMI), systolic and diastolic blood pressure (SBP, DBP), heart rate (HR), creatinine (CREA), uric acid (UA), random blood glucose (RBG), low-density lipoprotein cholesterol (LDL-C), estimated glomerular filtration rate (eGFR), hematocrit (HCT), neutrophil count (NEUT), lymphocyte count (LYMPH), neutrophil-to-lymphocyte ratio (NLR), hemoglobin (HGB), platelet count (PLT), C-reactive protein (CRP), myoglobin (MYO), creatine kinase-MB (CK-MB), troponin I (TNI), N-terminal pro B-type natriuretic peptide (NT-proBNP), hemoglobin A1c (HbA1c), left atrial diameter (LAD), left ventricular ejection fraction (LVEF), left ventricular end-diastolic and end-systolic volumes (LVEDV, LVESV), and medications including beta-blockers (BB), angiotensin-converting enzyme inhibitors (ACEI), angiotensin II receptor blockers (ARB), angiotensin receptor-neprilysin inhibitors (ARNI), sodium-glucose cotransporter-2 inhibitors (SGLT2i), statins (STAT), aspirin (ASA), ticagrelor (TICA), clopidogrel (CLOP), and proton pump inhibitors (PPI). Key clinical events such as ventricular fibrillation (VF), atrial fibrillation (AF), and cardiogenic shock (CS) were also documented.

### Model development and explanation

Patients were randomly divided into training and testing cohorts in a 7:3 ratio. Prior to model construction, several data processing steps were implemented: the synthetic minority oversampling technique (SMOTE) was applied to address class imbalance (Shi and Fan, 2023), Boruta was used for feature selection (Kursa and Rudnicki, 2010), and grid search with cross-validation (GSCV) was employed for hyperparameter tuning. Six ML algorithms were evaluated: RF, light gradient boosting machine (LightGBM), extreme gradient boosting (XGBoost), logistic regression (LR), k-nearest neighbors (KNN), and deep neural networks (DNN).

**Figure 1:**
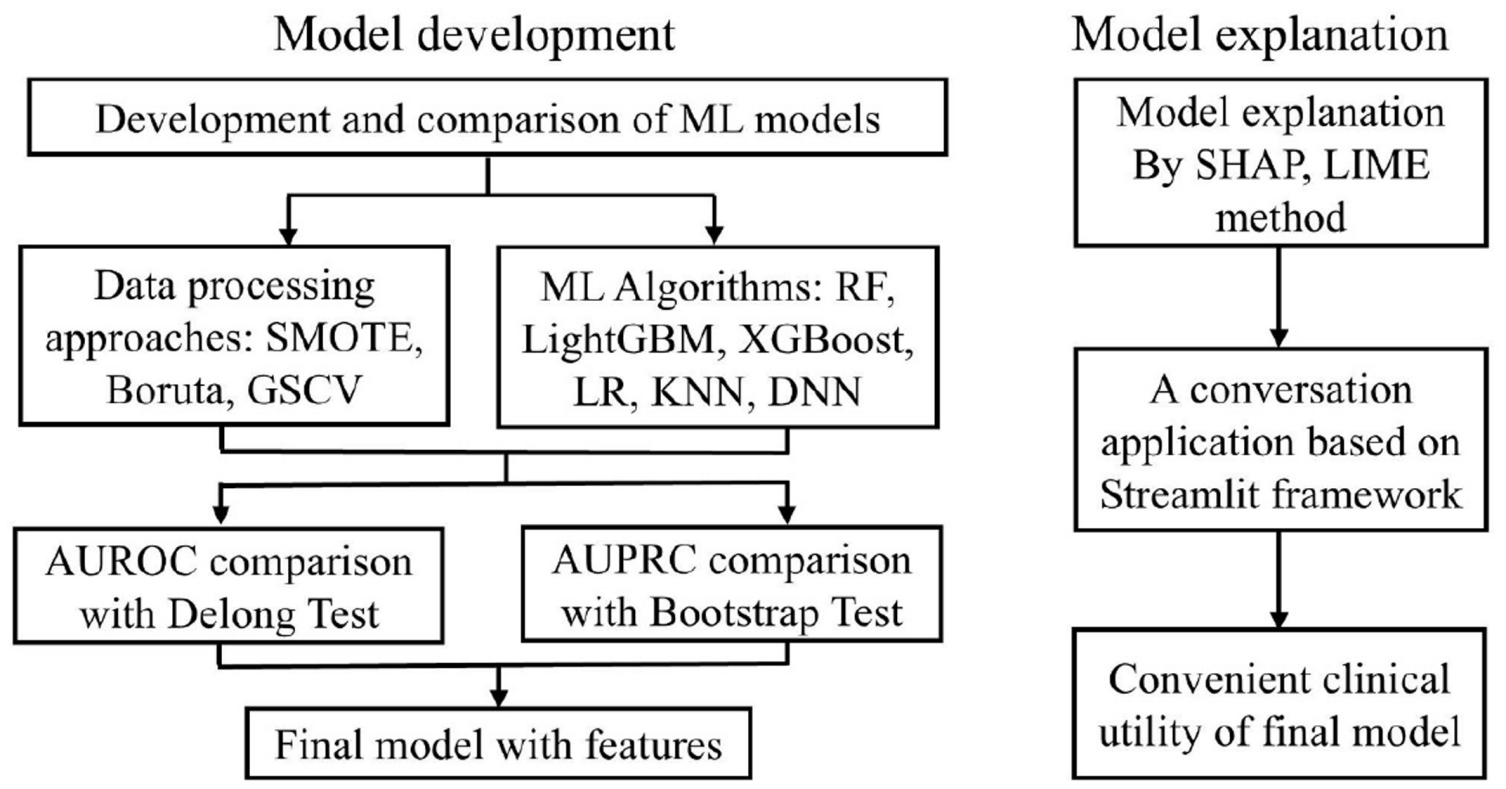
The ML model development process. ML, machine learning; SMOTE, synthetic minority over-sampling technique; GSCV, grid search with cross-validation; SHAP, shapley additive explanation; LIME, Local Interpretable Model-agnostic Explanations.

Model development adhered to a structured three-step framework: (1) identifying the optimal data processing pipeline, (2) selecting the best-performing ML algorithm, and (3) constructing the final predictive model by integrating the chosen processing and algorithmic strategies (Figure 1). Model performance was primarily assessed using AUROC and AUPRC (Zhou et al., 2021). Additional evaluation metrics—accuracy, precision, sensitivity, specificity, and F1-score—were employed to support model selection.

To enhance interpretability, SHAP and local interpretable model-agnostic explanations (LIME) analyses were employed to evaluate feature importance and quantify the contribution of individual variables to model predictions (Qi et al., 2025). SHAP force plots and LIME were utilized to visualize the influence of each feature on individual predictions, thereby improving transparency and supporting personalized clinical interpretation.

### Classes of risk

The pooled dataset, which includes 1,274 patients from both the derivation and external validation datasets, was categorized into low- and high-risk levels based on clinically meaningful thresholds. The stratification threshold was determined by analyzing the predicted probability distribution and calibration curves of the model in both the training and validation datasets. The model assesses individualized risk probabilities for specific patients based on input parameters and subsequently stratifies them into low-risk or high-risk groups using predefined thresholds. This risk stratification directly informs clinical decision-making regarding treatment intensity and follow-up frequency, thereby serving as a reference for personalized therapeutic strategies.

### Statistical analysis

All statistical analyses were conducted using R Software (version 4.3.1; http://www.r-project.org). The development of predictive models was carried out with Python (version 3.11.9; https://www.python.org) and PyCharm (version 2024.1.4). For normally distributed continuous variables, data are presented as means with standard deviations, while comparisons between groups were performed using independent sample t-tests. For variables with abnormal distributions, data are expressed as median values (Q1, Q3), and the Mann–Whitney U test was utilized for comparisons between two groups. Differences in AUROCs between models were assessed using DeLong’s test, and differences in AUPRCs were evaluated using the bootstrap method. Categorical variables are reported as counts (percentages) and were compared using the Chi-squared test. If the expected frequency of any cell was less than 5, Fisher’s exact test was employed. A two-tailed *p*-value of less than 0.05 was considered statistically significant. The Pearson correlation matrix was used to quantify the linear relationships among features.

## Results

### Population characteristics

A total of 672 patients were included in the derivation cohort for developing the one-year mortality prediction model, among whom 38 (5.7%) died within one-year post-PCI. Regarding missing data, BMI and HR were absent in 1 case (0.1%), HbA1c in 69 cases (10.3%), LAD in 8 cases (1.2%), LVEF in 6 cases (0.9%), LVEDV and LVESV in 7 cases (1.0%). The external validation cohort comprised 602 patients, with 21 (3.5%) experiencing one-year mortality. A comparative analysis of demographic and clinical characteristics between survivors and non-survivors is presented in Table 1, while Supplementary Table S1 provides a detailed comparison between the training and testing cohorts. Our analysis of baseline characteristics revealed statistically significant differences between the modeling cohort and the external validation cohort across multiple clinical variables, including medical history, vital signs, laboratory values, medication use patterns, and clinical outcomes (*p* < 0.05). However, within the modeling cohort, the training and validation subsets exhibited well-balanced characteristics without significant differences, thereby supporting the robustness of our internal validation approach.

### Model selection and performance comparison

**Figure 2:**
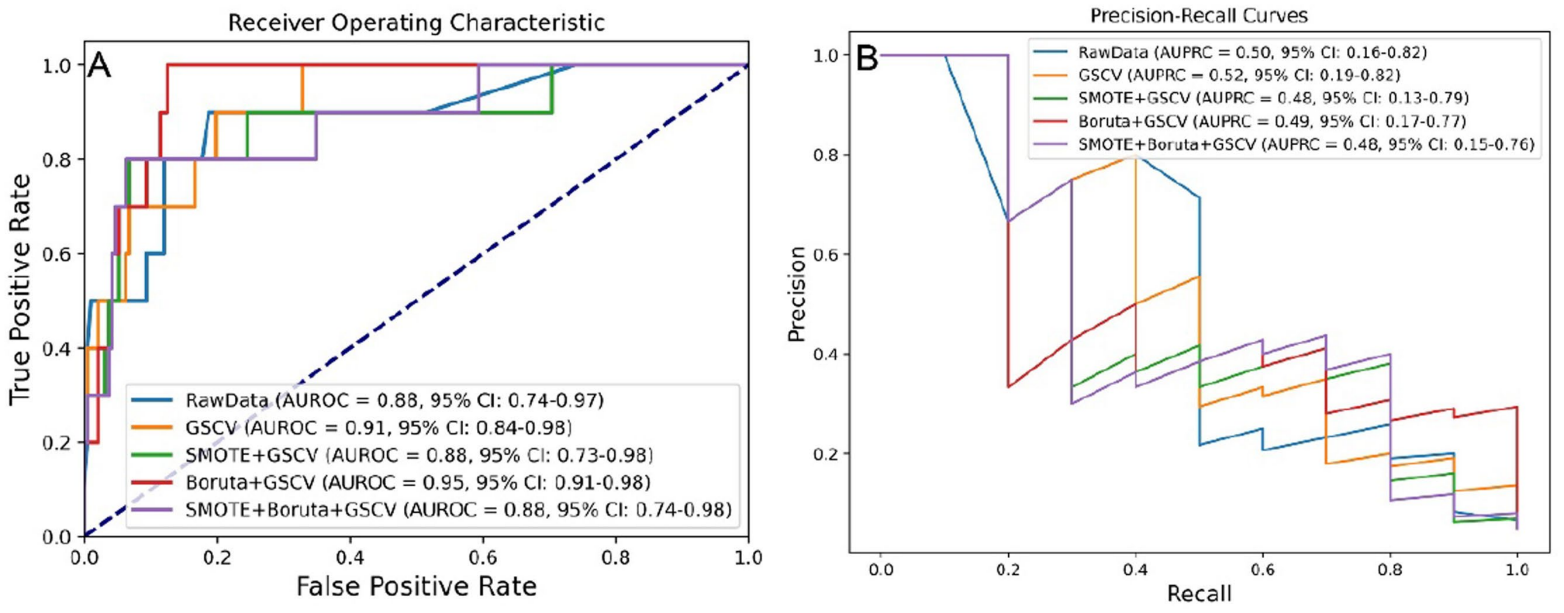
Model performance under five data preprocessing strategies. **(A)** AUROC values across different preprocessing levels. **(B)** AUPRC values across different preprocessing levels. SMOTE, synthetic minority over-sampling technique; GSCV, grid search with cross-validation; AUROC, area under the receiver operating characteristic curve; AUPRC, area under the precision-recall curve.

To optimize predictive performance, data processing methods—including SMOTE, Boruta, and GSCV—were applied where appropriate. The effects of these processing strategies were evaluated using the AUROC and AUPRC metrics, as illustrated in Figures 2A,B, respectively.

Although processing steps influenced model performance, the differences in AUROC were not statistically significant based on the DeLong test (Supplementary Figure S2A), nor were AUPRC differences significant according to bootstrap testing (Supplementary Figure S2B).

Key performance metrics—AUROC, AUPRC, accuracy, precision, sensitivity, specificity, and F1 score—were utilized to assess model efficacy. As shown in Supplementary Table S2, the model developed using Boruta and GSCV achieved an AUROC of 0.949, AUPRC of 0.486, accuracy of 0.950, precision of 0.500, sensitivity of 0.400, specificity of 0.979, and F1 score of 0.444.

**Figure 3:**
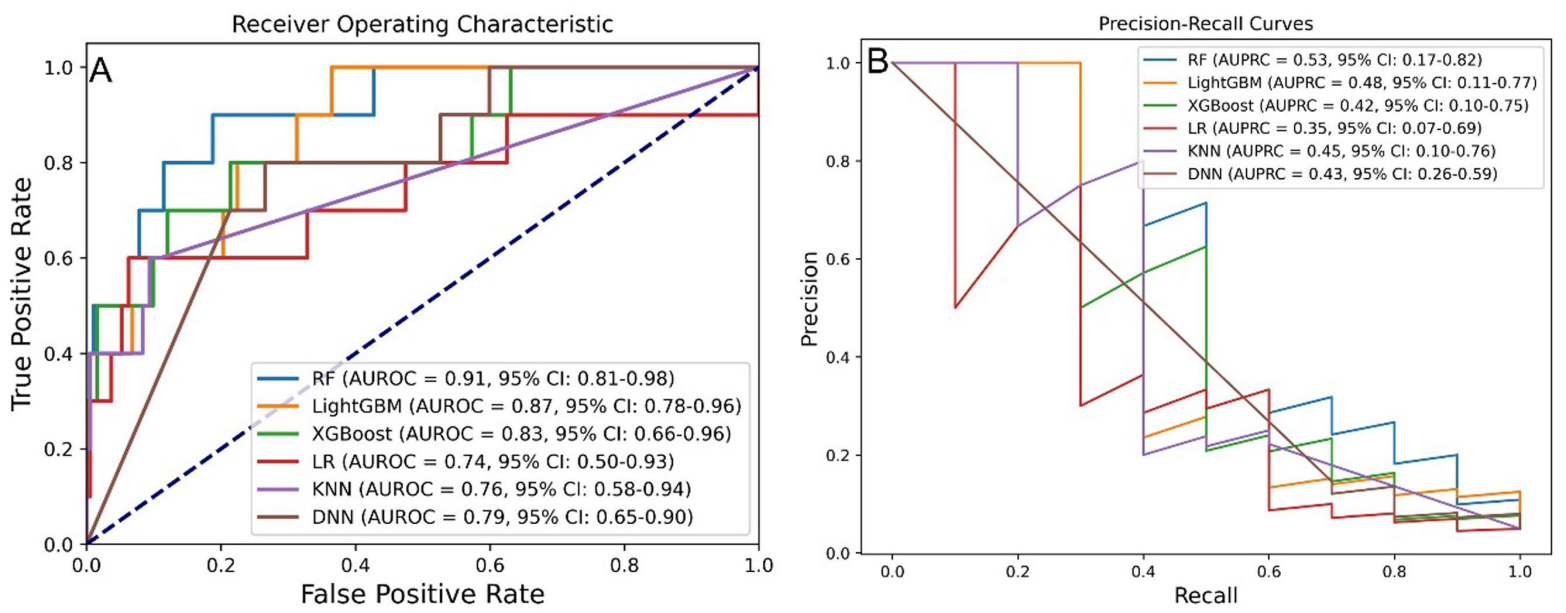
Model performance across six ML algorithms. **(A)** AUROC values for models developed using six algorithms. **(B)** AUPRC values for models developed using six algorithms. ML, machine learning; RF, random forest; LightGBM, light gradient boosting machine; XGBoost, extreme gradient boosting; LR, logistic regression; KNN, k-nearest neighbor; DNN, deep neural network; AUROC, area under the receiver operating characteristic curve; AUPRC, area under the precision-recall curve.

Six ML algorithms were evaluated, with the results for AUROC and AUPRC presented in Figures 3A,B. Among these algorithms, RF, LightGBM, and XGBoost exhibited the best predictive performance. The RF model achieving an AUROC of 0.91 (95% confidence interval (CI): 0.81–0.98) and an AUPRC of 0.53 (95% CI: 0.17–0.82).

Upon reevaluation of the impact of processing methods, it was determined that the differences in AUROC among the models were not statistically significant, as assessed by the DeLong test (Supplementary Figure S3A). However, the RF model displayed a significantly higher AUPRC compared to the LR model, while the differences in AUPRC among the other models remained non-significant based on bootstrap testing (Supplementary Figure S3B).

As summarized in Supplementary Table S3, the RF model exhibited superior overall performance, achieving an AUROC of 0.911, AUPRC of 0.534, accuracy of 0.960, precision of 0.667, sensitivity of 0.400, specificity of 0.990, and an F1 score of 0.500.

### Final model identification

No statistically significant differences in model performance (AUROC/AUPRC) were observed across various data processing strategies. Consequently, the method that offered the best balance between predictive precision and simplicity was selected. Among the evaluated ML algorithms, the RF model—optimized using Boruta and GSCV—achieved the highest AUROC and AUPRC values, thereby being designed as the optimal predictive model. Internal validation demonstrated an AUROC of 0.94 (95% CI: 0.90–0.98) and an AUPRC of 0.44 (95% CI: 0.15–0.76), while external validation yielded an AUROC of 0.93 (95% CI: 0.86–0.99) and an AUPRC of 0.70 (95% CI: 0.51–0.88) (Supplementary Figure S4).

To enhance clinical utility, patients were stratified into high- and low-risk groups using a probability threshold of 0.6. Calibration analysis (Supplementary Figure S5) demonstrated a systematic overestimation of high-risk predictions, while showing excellent agreement within the low-to-medium risk ranges, thereby supporting clinical reliability. Probability distributions by outcome (Supplementary Figure S6) exhibited a clear separation between survivors and non-survivors. Kernel density plots indicated significantly higher predicted probabilities for deceased patients (Mann–Whitney U test, *p* < 0.001), with the current threshold (*p* = 0.6, represented by the black dashed line) and the suggested optimal range (indicated by green shading). Boxplots further illustrated these distinct distributions.

**Figure 4:**
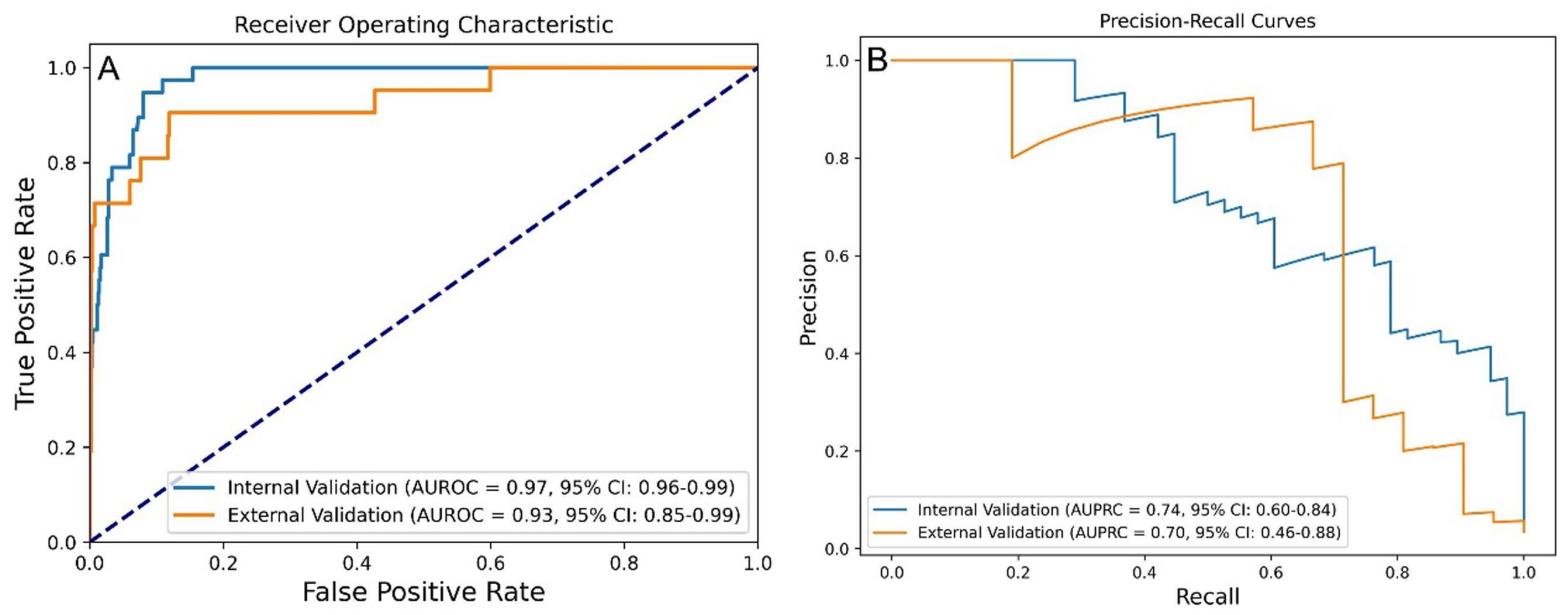
Performance of the stratified RF model in predicting one-year mortality among STEMI patients post-PCI. **(A)** AUROC values of stratified-RF model. **(B)** AUPRC values of the stratified-RF model. STEMI, ST-segment elevation myocardial infarction; RF, random forest; PCI, percutaneous coronary intervention; AUROC, area under the receiver operating characteristic curve; AUPRC, area under the precision-recall curve.

The post-stratification model performance is illustrated in Figure 4. Internal validation yielded an AUROC of 0.97 (95% CI: 0.96–0.99) and AUPRC of 0.74 (95% CI: 0.60–0.84), while external validation showed an AUROC of 0.93 (95% CI: 0.85–0.99) and AUPRC of 0.70 (95% CI: 0.46–0.88).

Performance metrics before and after risk stratification are summarized in Table 2. After stratification, the final model achieved an accuracy of 0.985, a precision of 0.875, a sensitivity of 0.667, a specificity of 0.997, and an F1 score of 0.757 in the external validation set.

**Table 2:** Performance of the final model after applying the risk stratification method.

Data processing	Accuracy	Precision	Sensitivity	Specificity	F1-score	AUROC	AUPRC
Internal validation							
Boruta + GSCV	0.950	0.500	0.400	0.979	0.444	0.942	0.443
Boruta + GSCV + Stratified	0.960	0.720	0.474	0.989	0.571	0.971	0.741
External validation							
Boruta + GSCV	0.953	0.405	0.714	0.962	0.517	0.932	0.699
Boruta + GSCV + Stratified	0.985	0.875	0.667	0.997	0.757	0.929	0.699

AUROC, the area under the receiver operating characteristic curve; AUPRC, area under the precision-recall curve; GSCV, grid search with cross-validation; RF, random forest.

### Model development

**Figure 5:**
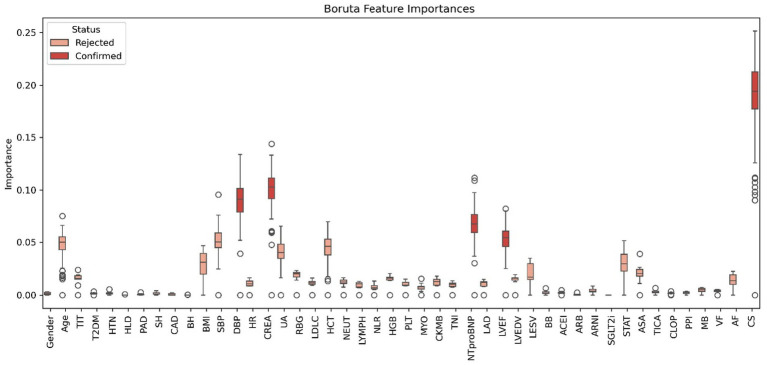
Features chosen in the training cohort using the Boruta algorithm. TIT, total ischemic time; CAD, coronary artery disease; HTN, hypertension; T2DM, type 2 diabetes mellitus; HLD, hyperlipidemia; PAD, peripheral arterial disease; SH, smoking history; BH, bleeding history; BMI, body mass index; SBP, systolic blood pressure; DBP, diastolic blood pressure; HR, heart rate; CREA, serum creatinine; UA, uric acid; RBG, random blood glucose; LDL-C, low-density lipoprotein cholesterol; eGFR, estimated glomerular filtration rate; HCT, hematocrit; NEUT, neutrophil count; LYMPH, lymphocyte count; NLR, neutrophil-to-lymphocyte ratio; HGB, hemoglobin; PLT, platelet count; CRP, c-reactive protein; MYO, myoglobin; CK-MB, creatine kinase-mb; TNI, troponin I; NT-proBNP, n-terminal pro b-type natriuretic peptide; HbA1c, hemoglobin a1c; LAD, left atrial diameter; LVEF, left ventricular ejection fraction; LVEDV, left ventricular end-diastolic volume; LVESV, left ventricular end-systolic volume; BB, beta-blockers; ACEI, angiotensin-converting enzyme inhibitors; ARB, angiotensin II receptor blockers; ARNI, angiotensin receptor-neprilysin inhibitors; SGLT2i, sodium-glucose cotransporter-2 inhibitors; STAT, statins; ASA, aspirin; TICA, ticagrelor; CLOP, clopidogrel; PPI, proton pump inhibitors; VF, ventricular fibrillation; AF, atrial fibrillation; CS, cardiogenic shock.

The application of the Boruta algorithm for feature selection significantly enhanced model interpretability and mitigated overfitting by reducing the input set from 46 features to just 5. This reduction not only preserved the model’s performance but also simplified its implementation. Figure 5 illustrates the features selected by the Boruta algorithm. Furthermore, the optimized RF model was configured with the following hyperparameters: a maximum tree depth of 30, a minimum of samples per split, a total of 300 decision trees.

### Model explanation

**Figure 6:**
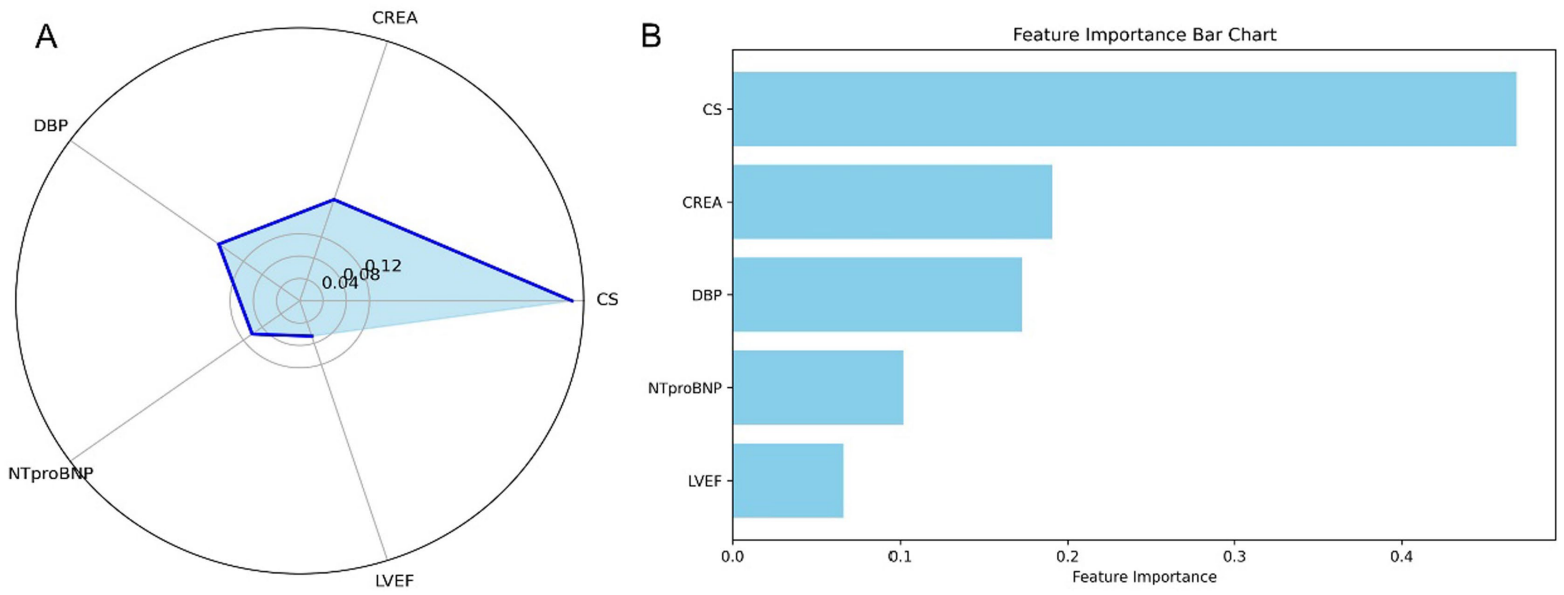
Feature importance in the stratified RF model for predicting one-year mortality in STEMI patients post-PCI. **(A)** Radar plot depicting feature importance for the stratified RF model. **(B)** SHAP summary bar plot illustrating the contributions of individual features to the model’s predictions. STEMI, ST-segment elevation myocardial infarction; RF, random forest; PCI, percutaneous coronary intervention; CS, cardiogenic shock; CREA, creatinine; NT-proBNP, N-terminal pro-B-type natriuretic peptide; DBP, diastolic blood pressure; LVEF, left ventricular ejection fraction.

We employed the Pearson correlation matrix to assess multicollinearity among all modeling features (Supplementary Figure S7). The results indicated four pairs of features exhibiting high collinearity (|r| > 0.7): SBP and DBP (r = 0.75), HCT and HGB (r = 0.95), CKMB and TNI (r = 0.83), and LVEDV and LESV (r = 0.91). These strong correlations suggest potential redundancy, emphasizing the need for dimensionality reduction or variable selection to mitigate multicollinearity during model development. The final model did not include the afore mentioned features simultaneously. Feature importance in the final RF model is illustrated in a radar plot (Figure 6A) and a SHAP summary bar plot (Figure 6B), highlighting the five variables that influence one-year mortality. The model comprises five features, ranked by weight from highest to lowest: CS (0.433), CREA (0.200), DBP (0.191), NT-proBNP (0.104), and LVEF (0.072).

**Figure 7:**
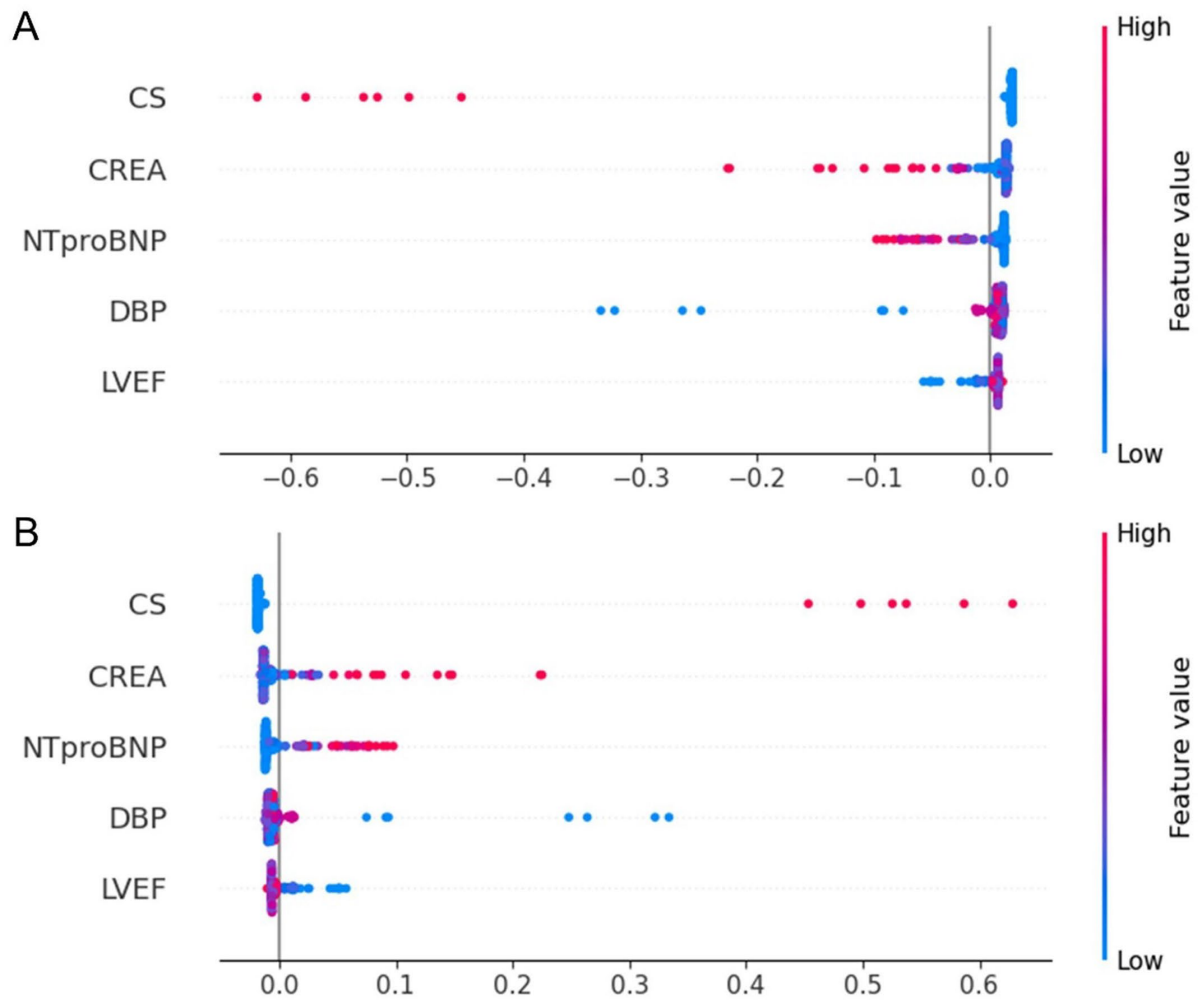
SHAP summary dot plot for the stratified RF model predicting one-year mortality in STEMI patients post-PCI. **(A)** SHAP summary plot for the model predicting death as a positive outcome. **(B)** SHAP summary plot for the model predicting survival as a positive outcome. The plot illustrates the contribution of each feature to the model’s predictions. STEMI, ST-segment elevation myocardial infarction; RF, random forest; PCI, percutaneous coronary intervention; CS, cardiogenic shock; CREA, creatinine; NT-proBNP, N-terminal pro–B-type natriuretic peptide; DBP, diastolic blood pressure; LVEF, left ventricular ejection fraction.

**Figure 8:**
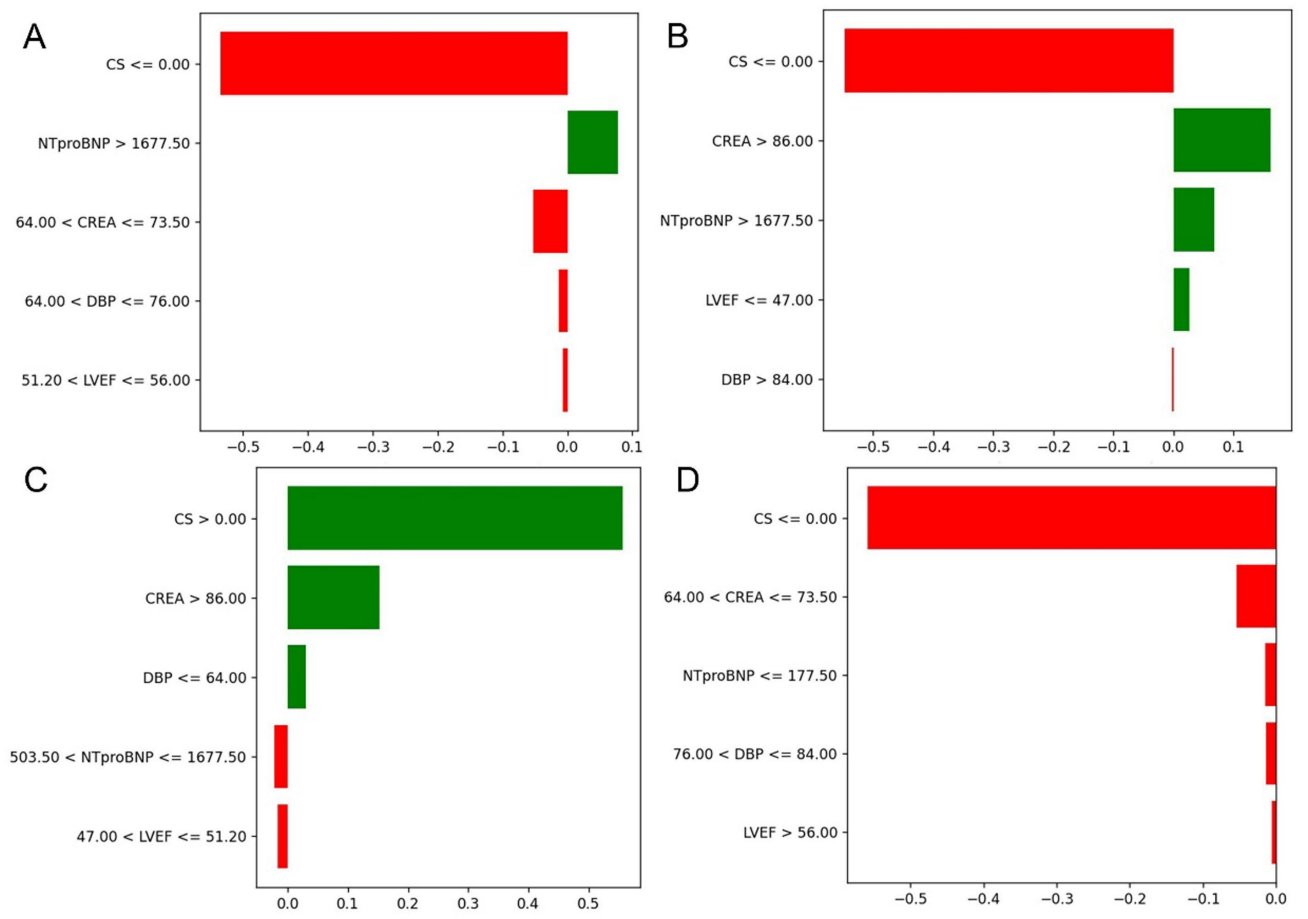
Contribution of key clinical features to individual risk prediction across subgroups. **(A–D)** Illustrate the directional impact of selected discretized features on model output across different patient strata. The horizontal bars represent both the magnitude and direction of contribution for each feature, with red indicating a negative (risk-reducing) effect and green indicating a positive (risk-enhancing) effect.

To enhance transparency and clinical interpretability, SHAP was used to analyze overall feature importance, while LIME was employed to examine local feature weights and decision rules for individual samples. Figure 7 shows SHAP summary plots ranking feature importance based on mean SHAP values. The most influential predictors included CS, CREA, NT-proBNP, DBP, and LVEF. Figure 8 illustrates the contribution of key clinical features to individual risk prediction across various patient subgroups. Notably, across all subgroups, the absence of cardiogenic shock (CS ≤ 0) consistently emerged as the strongest negative predictor. Elevated levels of NTproBNP (>1677.5 pg./mL) and impaired renal function (CREA >86 μmol/L) were identified as significant risk enhancers. Additionally, subtle variations in diastolic blood pressure (DBP) and left ventricular ejection fraction (LVEF) contributed to risk modulation specific to each subgroup.

### Clinical utility and application

**Figure 9:**
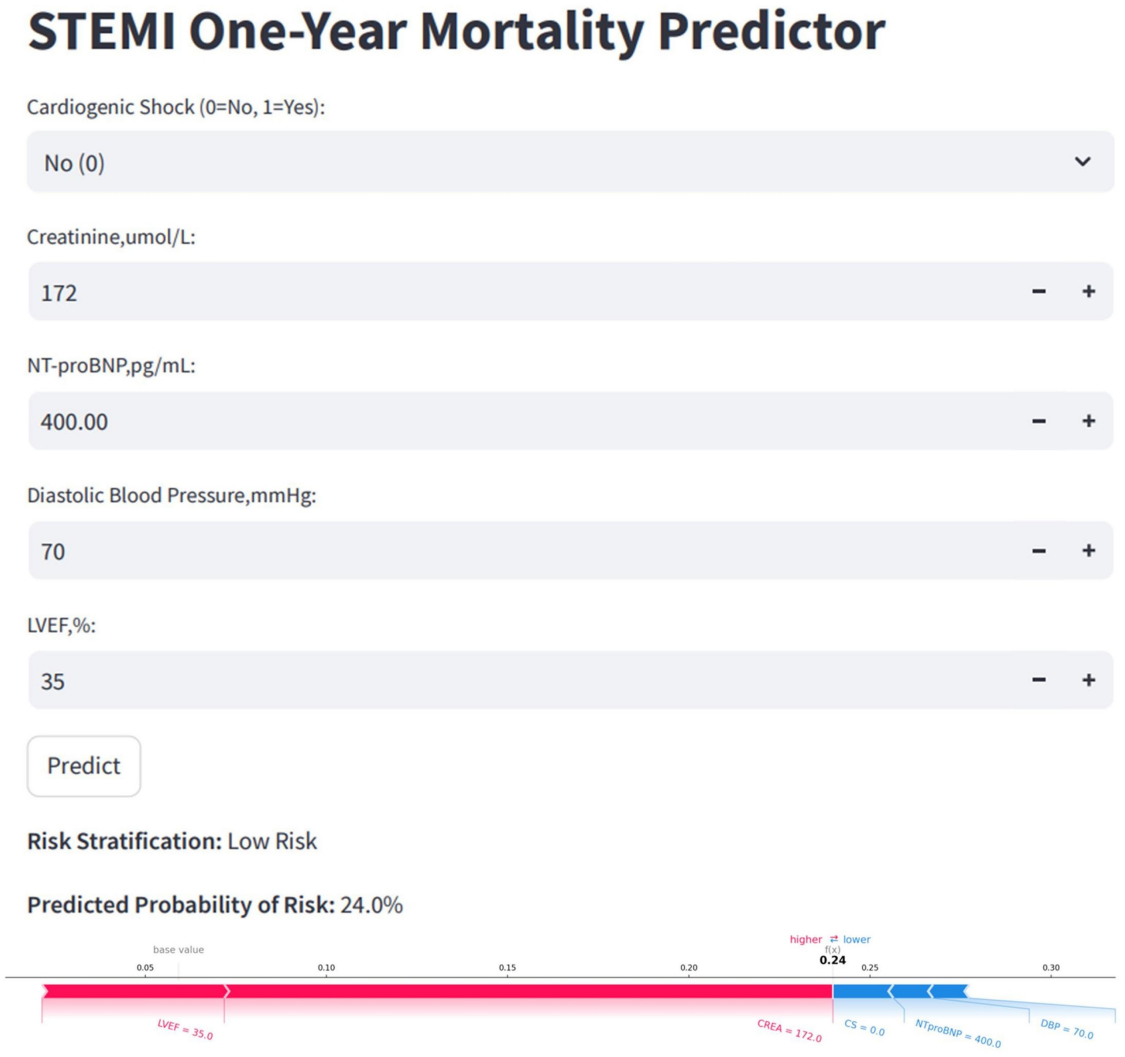
Convenient application for clinical utility. The convenient application of the stratified RF model with 5 features is available for STEMI one-year mortality prediction. When entering actual values of the 5 features, this application automatically displays the probability of 24%. Meanwhile, the force plot for individual child indicates the features that contribute to the decision of mortality: the blue features on the right are the features pushing the prediction towards decease, while the red features on the left are pushing the prediction towards survival. RF, random forest; STEMI, st-segment elevation myocardial infarction.

To facilitate clinical application, the final model was deployed as a web-based tool (Figure 9). Clinicians can input patient values for the five selected features, and the tool will automatically calculate the predicted risk of one-year mortality for STEMI patients post-PCI. Additionally, it generates a personalized SHAP force plot that visually highlights the factors influencing each prediction. In these plots, the blue features on the right indicate variables associated with improved survival, while the red features on the left represent factors that contribute to an increased risk of mortality.

## Discussion

This study successfully developed a predictive model for estimating the 1-year mortality risk in STEMI patients following PCI and validated a systematic approach to model construction. Based on the characteristics of the raw data, appropriate processing strategies and ML algorithms were selected. Model performance was evaluated using AUROC and AUPRC metrics. The final streamlined model included only five clinical variables: CS, CREA, NT-proBNP, DBP, and LVEF. Among all candidate models, the one constructed using the Boruta, GSCV, and the RF algorithm demonstrated the best performance, with an AUROC of 0.94 and an AUPRC of 0.44 in the internal validation set. However, the application of risk stratification substantially improved model performance, achieving an AUROC of 0.97 and an AUPRC of 0.74. We propose categorizing patients into two risk classes (low and high) for each outcome. This stratification aims to underscore the clinical implications of each risk value computed by the model. By selecting a relatively high threshold, we corrected the overestimated event rates in the overall test set, thereby enhancing model performance. This approach ensured stability within specific populations and mitigated inflated metrics caused by risk overestimation. Following stratification, the sample characteristics within high and low-risk groups became more homogeneous, thereby improving the model’s predictive accuracy within each subgroup. These findings suggest that risk stratification is an effective method for enhancing model performance in imbalanced datasets.

Clinical databases often exhibit missing values, high dimensionality, and class imbalance, necessitating robust processing strategies (Mohammadi et al., 2023; Chan et al., 2023; Iacobescu et al., 2024; Hosseini et al., 2024; Öztekin and Özyılmaz, 2025). Prior studies have addressed these challenges using techniques such as SMOTE and GSCV (Oliveira et al., 2023). For instance, Iacobescu et al. (2024) utilized SMOTE and GSCV to enhance model performance in a highly imbalanced dataset (91.91% vs. 8.09%); however, they did not quantify the impact of processing. Hosseini et al. (2024) analyzed 9,073 AMI patients and reported an AUROC of 0.866 for the RF model following the application of SMOTE. Similarly, Öztekin and Özyılmaz (2025) employed SMOTE with 38 features to develop an in-hospital mortality model, achieving high predictive performance, despite a small sample size. Nonetheless, these studies did not evaluate the statistical significance of processing on model performance or delineate when SMOTE is necessary. In our study, we systematically assessed various processing strategies and confirmed, using DeLong’s test, that there were no significant differences in AUROC. Consequently, we selected the simplest effective approach to maximize model interpretability and clinical relevance.

Choosing the most suitable ML algorithm remains a significant challenge in predictive modeling (Yang et al., 2025; Liu et al., 2022; Jeong et al., 2024). Most studies recommend algorithm based on conventional metrics—such as AUROC, F1-score, precision, sensitivity, and accuracy—yet few assess the statistical significance between models (Zhang et al., 2023; Zheng et al., 2023). For instance, D'Ascenzo et al. (2021) developed the PRAISE score for one-year mortality in ACS patients using adaptive boosting (Adaboost), naive bayes, KNN, and RF, concluding that Adaboost performed the best. Khera et al. (2021) compared four models (XGBoost, neural networks, meta-classifier, and LR) for predicting in-hospital AMI mortality, finding no significant improvement of ML models over LR, with all AUPRC values below 0.4. Although these comparisons are informative, they lack statistical testing. Notably, Hu et al. (2024) employed the DeLong test to evaluate algorithmic differences, thereby adding methodological rigor. In our study, we compared RF, LightGBM, XGBoost, LR, KNN, and DNN. Ensemble methods like RF reduce variance through bagging, while LightGBM and XGBoost enhance accuracy via boosting and regularization. LR provides interpretability, KNN offers flexibility with structured data, and DNNs excel in handling complex, high-dimensional data. Our findings support the growing consensus that there is no universally best algorithm—only the most contextually appropriate one. Despite algorithmic advances, improvements are not always statistically significant, as confirmed by our results.

While increasing the number of input features may initially enhance model performance, the improvements often plateau (Chen et al., 2024; Hamilton et al., 2024; Li et al., 2024; Liu et al., 2024; Hu et al., 2024). Therefore, constructing models with a limited number of clinically accessible features becomes a primary objective. Some researchers employ LASSO or SHAP to rank feature importance and manually select the most significant variables. For example, Hu et al. (2024) successfully reduced 33 features to 8 using SHAP without a notable decline in performance. Similarly, Liu et al. (2024) developed a readmission model for NSTEMI patients, condensing 96 features to 7 through the use of LR, RF, and LASSO, ultimately finding the LR-based model to be the most effective. However, these studies generally did not evaluate the statistical significance of performance differences between models, which limits their robustness.

Recognizing the complexity of real-world clinical data, we emphasize that simplicity and usability are critical for the adoption of models. Consequently, we employed DeLong and bootstrap tests to compare the AUROC and AUPRC across various models, ultimately finding no statistically significant differences. Based on these results, we selected the simplest model capable of reliably identifying positive cases, which aligns with the priorities of clinical decision-making.

In addition to limitations related to sample size and data quality, we contend that the reliance on static variables to predict dynamic clinical outcomes is inherently restrictive. Future advancements in real-time, dynamic prediction models—incorporating time-series analysis and multimodal data integration—may offer a more precise and personalized approach to risk stratification and clinical decision-making (Ren et al., 2025).

### Limitations

There are several limitations to our study. First, as a single-center, retrospective investigation with a limited sample size, it is essential to conduct multicenter, prospective studies for more robust findings. Second, although we performed external validation, all data were sourced from the same hospital, which may introduce selection bias and lacks validation across diverse institutional settings. Third, the clinical utility of this prognostic prediction model is constrained by its inability to incorporate time-series analysis and integrate multimodal data, which limits its applicability in real-world scenarios.

## Conclusion and future work

In conclusion, we developed an RF-based predictive model utilizing Boruta for feature selection and GSCV for optimization, effectively reducing dimensionality and enhancing model performance in predicting one-year mortality in STEMI patients post-PCI. Additionally, integrating a risk stratification approach significantly improved the model’s clinical applicability.

This work highlights several gaps that must be addressed in future research before implementing this type of model construction. First, while SMOTE is an effective method for addressing class imbalance, particularly when the imbalance ratio exceeds 10:1, it may overlook intra-class distributions. Additionally, its interpolation of minority-class samples can lead to the generation of noisy instances, potentially increasing model complexity and degrading performance. SMOTE is most effective when the data is imbalanced and the minority class exhibits clear distribution patterns. Second, although SHAP provides valuable insights into feature contributions, its reliability may be compromised in the presence of strong feature multicollinearity. Future work should incorporate correlation analysis and feature selection or reduction prior to interpretation to ensure more coherent SHAP results.

## Data Availability

The original contributions presented in the study are included in the article/supplementary material, further inquiries can be directed to the corresponding author.
